# Rupture of liver metastasis in high‐volume metastatic prostate cancer patient on androgen deprivation therapy combined with upfront docetaxel chemotherapy

**DOI:** 10.1002/iju5.12512

**Published:** 2022-07-27

**Authors:** Kenichi Hata, Masatoshi Tanaka, Kazuhiro Takahashi, Takahiro Kimura

**Affiliations:** ^1^ Department of Urology Atsugi City Hospital Atsugi City Japan; ^2^ Department of Urology Jikei University School of Medicine Tokyo Japan

**Keywords:** androgen deprivation, docetaxel, hepatorrhexis, liver metastasis, prostate cancer

## Abstract

**Introduction:**

Recent studies have indicated an improvement in the survival rate of patients using docetaxel in addition to androgen deprivation therapy for high‐volume metastatic hormone‐sensitive prostate cancer. Hepatorrhexis, characterized by the rupture of the liver, is a rare complication associated with chemotherapies. We report a case of hepatic metastases rupture during androgen deprivation therapy combined with upfront docetaxel chemotherapy.

**Case presentation:**

A 77‐year‐old man diagnosed with high‐volume metastatic hormone‐sensitive prostate cancer received an upfront docetaxel treatment combined with androgen deprivation therapy. Hepatic metastases rupture with substantial hemoperitoneum occurred on the 14th day of the fifth cycle of docetaxel chemotherapy. Transcatheter arterial embolization was performed; however, despite receiving optimal supportive care, the patient died.

**Conclusion:**

The addition of upfront docetaxel to androgen deprivation therapy may be effective in patients with high‐volume metastatic hormone‐sensitive prostate cancer; however, strict observation is required to monitor for the occurrence of rare complications, including hepatorrhexis.

**Abbreviations & Acronyms**
ADT: androgen deprivation therapy
HCC: hepatocellular carcinoma
mHSPC: metastatic hormone‐sensitive prostate cancer
PSA: prostate‐specific antigen
TAE: transcatheter arterial embolization

**Keynote message**

We observed a rare case of the rupture of hepatic metastases in a patient receiving docetaxel and ADT for high‐volume mHSPC.

## Introduction

Androgen deprivation therapy (ADT) has remained the mainstay of treatment for patients with metastatic hormone‐sensitive prostate cancer (mHSPC) for more than 70 years.^1^ Although initially effective, castration‐resistance eventually develop in almost all patients with advanced prostate cancer.^2^ Recent studies from three large, randomized, phase 3 clinical trials have indicated longer overall survival periods in patients receiving upfront docetaxel chemotherapy plus ADT for high‐volume mHSPC.^3^, ^4^, ^5^ Moreover, additional therapeutic regimens like abiraterone, apalutamide, and enzalutamide were also shown to have similar clinical effectiveness as upfront intensive therapy. These newer chemotherapeutic regimens have now become the standard treatment for patients with mHSPC.^6^

Spontaneous rupture (or hepatorrhexis) of a liver metastasis is a very rare event when compared with hepatocellular carcinoma (HCC). It occurs suddenly and can have potentially fatal outcomes. Both benign as well as malignant tumors of the liver have been cited as those among the most common causes of liver ruptures with HCC being cited as the leading cause of liver ruptures.^6^ Metastatic liver tumors from genitourinary disease, however, seem to be even more rare.^7^ We herein describe a rupture of a hepatic metastases that results as an adverse effect of ADT in combination with upfront docetaxel chemotherapy in a patient with high‐volume mHSPC.

## Case presentation

**Fig. 1:**
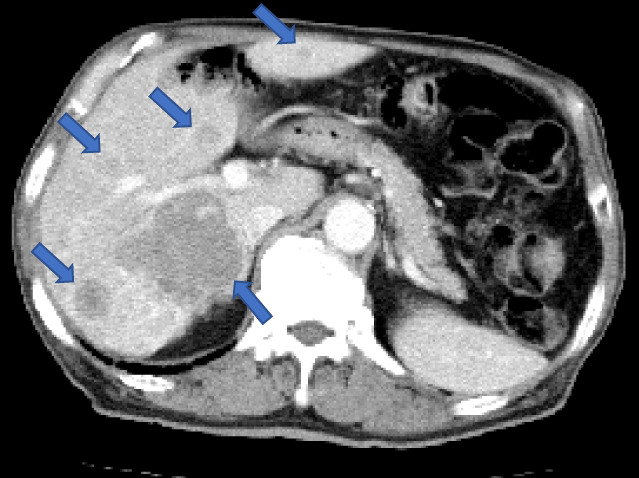
Enhanced computed tomography at diagnosis showing multiple liver metastases. The largest liver metastasis invaded the hepatic capsule with hepatic surface discontinuity.

**Fig. 2:**
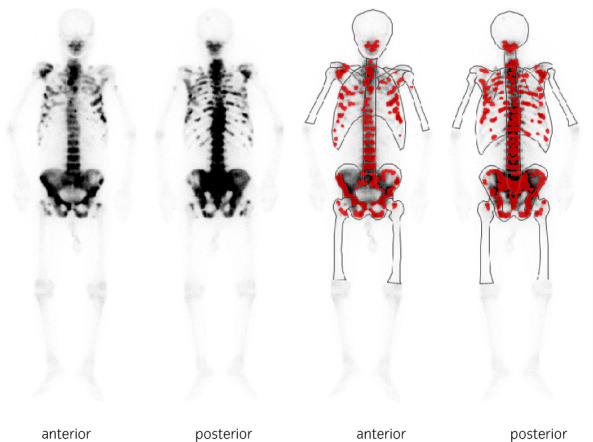
Bone scintigraphy at the time of diagnosis showing high volume multiple bone metastases.

A 77‐year‐old man was referred to our hospital with clinical conditions such as high alkaline phosphatase (1,1076 U/L) and prostate‐specific antigen (PSA, 783 ng/mL) levels, persistent fatigue, and a 3 month history of both appetite and weight loss. Histological examination of transrectal prostate biopsy confirmed the presence of Gleason grade 5 prostate cancer.^8^ Moreover, computed tomography revealed multiple liver (maximum size: 6.6 cm in diameter) and lymph node metastases. The largest liver metastasis invaded the hepatic capsule with hepatic surface discontinuity. Bone scintigraphy confirmed high‐volume bone metastases (Figs. 1, 2).^4^

**Fig. 3:**
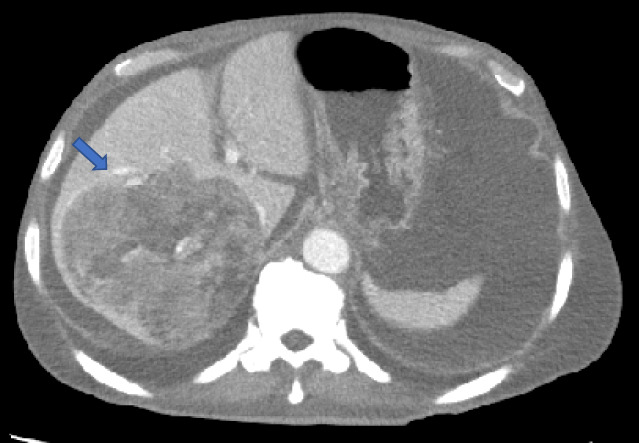
Enhanced computed tomography revealing hepatic metastases rupture with substantial hemoperitoneum hematemesis on the 14th day of the fifth course of upfront docetaxel.

**Fig. 4:**
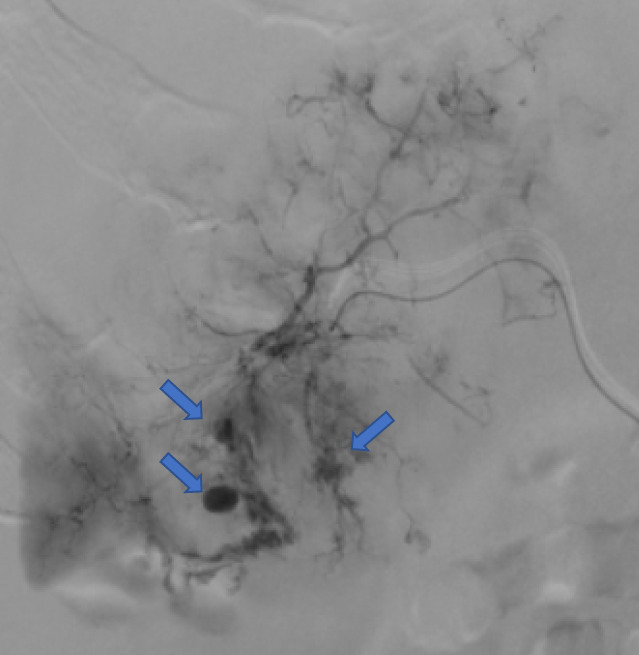
Selective arteriography of the right hepatic branch showing rupture of hepatic metastases. The arrows indicate rhexis of the hepatic artery.

After 2 weeks of hormonal therapy with goserelin acetate (Zoladex, Astrazeneca, Cambridge, UK) and bicalutamide (Casodex, Astrazeneca), the patient received upfront docetaxel (Taxotere, Sanofi, Paris, France) every 4 weeks for six cycles. He experienced mild fatigue, and there were no findings of bone marrow dysfunction during the first four cycles of docetaxel. After four cycles, the patient's PSA levels reduced to 14.45 ng/mL. On the 14th day of the fifth course of docetaxel, the patient was admitted to the emergency section on account of hematemesis, tarry stools, and hemodynamic shock. His hemoglobin levels rapidly dropped from 6.2 g/dL to 2.3 g/dL within an hour; he received eight units of red blood cells. Enhanced computed tomography revealed rupture of hepatic metastases with substantial hemoperitoneum while blood tests indicated deteriorating liver function (Fig. 3). Transcatheter arterial embolization (TAE) of the right hepatic branch using gelfoam particles was performed without delay (Fig. 4). Although vital signs stabilized after TAE, hepatic dysfunction progressed rapidly. The clinical data of the patient on the following day was as follows: bilirubin, 1.7 mg/dL; aspartate aminotransferase, 1094 IU/L; alanine aminotransferase, 238 IU/L; lactate dehydrogenase, 3,294 IU/L; alkaline phosphatase, 1,152 IU/L; gamma‐glutamyl transpeptidase, 418 IU/L; and white blood cell count, 71.9 × 10^9^/L. The patient was discontinued for the last docetaxel course. Despite receiving optimal care, the patient died of general prostration 3 weeks later, which occurred as an adverse event of ADT combined with upfront docetaxel. An autopsy was not performed per the family's request.

## Discussion

The spontaneous rupture of liver metastasis is a life‐threatening event in patients with various types of malignancies. Recent studies have indicated that primary tumors of lung, pancreas, skin, stomach, kidney, testis and prostate may result in secondary liver metastases and its subsequent rupture.^9^ Till date, no study has yet shown a correlation between prostate cancer and hepatorrhexis, although two cases of testicular and renal cancer have been reported.^10^, ^11^, ^12^, ^13^, ^14^ To our knowledge, the present study is the first report describing spontaneous rupture of liver metastasis caused by mHSPC. It is pertinent to mention that among mHSPC cases, liver metastasis occurred only in 1–5% of the cases^2^, ^3^, ^4^, ^5^ reflecting the rarity of spontaneous rupture of liver in mHSPC as compared to HCC‐related liver ruptures that occur in 4.8–26% of the cases.^7^, ^15^

The risk factors of hepatic metastasis rupture include necrotic tendency, a subcapsular location, and a rapid tumor growth.^16^, ^17^ Moreover, tumor necrosis resulting from chemotherapy has also been cited as a possible risk factor for metastatic rupture.^9^ We report that although chemotherapy was effective in reducing PSA levels, no significant change was observed in the size of multiple, bulky metastatic hepatic tumors. These finding indicated that hepatorrhexis may be caused when a considerable section of liver metastases changes necrotic tissue. In contrast, Xia et al. suggested that tumor diameter >5–7 cm; tumor protruding to >1 cm from the liver surface; and tumor located in segments II, III, VI B, and VI are independent risk factors for spontaneous rupture and hemorrhage of HCC.^18^ Most patients with HCC have a long history of cirrhosis, which leads to coagulation dysfunction, and hepatitis virus infection leads to destruction of the normal structure of the vascular walls of the liver through local inflammatory reactions.^18^ In our case, the largest liver metastasis in segment VI had a diameter of 6.6 cm and invaded the hepatic capsule. Hence, these factors might be associated with hepatorrhexis.

The role of TAE is well established in HCC rupture and there are case reports of its successful use in ruptured liver metastases.^7^ Despite the patient's poor clinical status, we were able to temporarily stabilize his hemodynamic parameters after TAE. Previous studies have reported successful TAE for metastatic liver rupture associated with various forms of cancer.^15^, ^16^, ^17^ Mochimaru *et al* reviewed successful TAE in two out of 11 cases in which rupture of liver metastasis was observed in patients with lung cancer during chemotherapy, and the patients survived for a long period.^16^ In contrast, two of the cases that underwent emergency operation died as a result, and the other seven cases, who were given conservative care only, died within a few months.^16^ In the present study, we evaluated the role of TAE to reduce the risk of lethal re‐bleeding. Our studies indicated that our patient's vital signs stabilized soon after TAE without re‐bleeding, but he died from general prostration three weeks later. Further studies are required to determine the therapeutic role of TAE in this setting.

Docetaxel chemotherapy was the first agent to demonstrate an improvement in the survival rate of men with metastatic castration resistant prostate cancer based on two landmark phase III trials TAX327 and before upfront docetaxel approval.^19^ Therefore, docetaxel has been widely used and a familiar toxicity profile of not only mHSPC but also metastatic castration resistant prostate cancer for physicians. However, Sweeny *et al* reported a case of sudden fatality in patient with an unknown cause.^4^ Moreover, five docetaxel‐related deaths in patients who had metastatic castration resistant prostate cancer were described in TAX 327 study.^19^ Our report presented a case of ruptured liver metastases during upfront docetaxel for high‐volume mHSPC following death. Strict observation during upfront docetaxel chemotherapy for patients with high‐volume mHSPC and bulky liver metastases invading the hepatic capsule, risk factors of hepatorrhexis, is important.

## Author contributions

Kenichi Hata: Conceptualization; data curation; formal analysis; investigation; methodology; project administration; writing – original draft. Masatoshi Tanaka: Data curation. Kazuhiro Takahashi: Data curation; writing – review and editing. Takahiro Kimura: Supervision; writing – review and editing.

## Ethical statement

An Institutional review board approval was not required as this was a single case. The patient provided informed consent.

## Conflicts of interest

Takahiro Kimura is a paid consultant/advisor of Astellas, Bayer, Janssen, and Sanofi. The other authors declare no conflicts of interest associated with this manuscript.

## Registry and the Registration No. of the study/trial

Not applicable.

## Data Availability

The authors confirm that the data supporting the findings of this study are available within the article.
